# COVID-19 related disruption in higher education students' health and wellbeing: Implications for university action

**DOI:** 10.3389/fpubh.2022.1015352

**Published:** 2022-09-15

**Authors:** Christiane Stock, Stefanie M. Helmer, Katherina Heinrichs

**Affiliations:** ^1^Charité – Universitätsmedizin Berlin, Corporate Member of Freie Universität Berlin and Humboldt-Universität zu Berlin, Institute of Health and Nursing Science, Berlin, Germany; ^2^Unit for Health Promotion Research, University of Southern Denmark, Esbjerg, Denmark; ^3^Human and Health Sciences, University of Bremen, Bremen, Germany

**Keywords:** university students, higher education, COVID-19, health promotion, wellbeing

## Abstract

The COVID 19 pandemic has led to substantial disruptions in the lives of students in higher education. Partial or full closures of institutions for face-to-face teaching or consultations over a long-lasting period of time in many countries have had significant consequences on the psychosocial health and wellbeing of many students. This perspectives article summarizes the implications on mental, social, and behavioral aspects of students' wellbeing. Moreover, the need for health promoting und supportive services, programmes, structures, and policies is outlined with recommendations for institutional actions on the level of teaching practice, counseling services, and health monitoring, and with the call to re-affirm the wider policy-oriented approach of the Health Promoting University.

## Introduction

The life of students in higher education changed drastically since spring 2020 due to the pandemic-related switch to online teaching and other pandemic-related restrictions (1). Educational institutions were temporarily closed in many countries worldwide, and distance learning was introduced with often very short notice. In many cases, the technical requirements were not even in place. Essential practice elements of curricula (e.g., laboratory work, sports, arts, or internships) were no longer applicable, and teaching methods requiring direct contact with or between students and relying on interpersonal interaction were strongly affected. Some practice-based teaching content has even become completely impossible. Since this change to online teaching was unplanned and required creative problem solving it is also termed as “Emergency Remote Teaching” (2).

Students had to familiarize themselves with the digital learning tools and platforms and adapt to the often modified or new teaching content and methods. They mostly worked in isolation at home, any group-based learning only took place virtually, and contact with the teaching staff was limited to emails and video chats. This disruption in students' life resulted in more difficulties in managing studies, less motivation, less engagement with their studies as well as the feeling of social isolation, and impairments of psychological wellbeing and mental health (1, 3).

Based on this background, this perspective article will summarize some implications of this disruption of students' lives on their psychosocial health and wellbeing and on their health risk behavior. This will further lead to recommendations and suggestions with respect to how universities and colleges could best deal with the consequences of the COVID-19 pandemic from our perspective.

## Implications for students' psychosocial health and wellbeing

Several studies report high stress levels among higher education students during the COVID-19 pandemic with negative consequences for academic performance and the general state of mental health (1, 4, 5). Higher education students have been described as being at high risk for mental health problems and difficulties even before the pandemic (6).

Related to the enhanced overall level of stress that has been experienced by university students during COVID-19, research reported increased financial and psychological stress in Australian medical students compared to pre-pandemic data (7). Other research in many countries has shown that students experienced anxiety due to COVID-19, including worries about economic difficulties, academic delays, and effects of COVID-19 on daily life (8–10). During the pandemic, the prevalence for depressive symptoms among medical students varied between 31 and 38%, as recent systematic reviews reported (11, 12). About every third medical student showed symptoms of anxiety (11), this rate being lower, though, than the prevalence found among overall students (41%) (13).

### Vulnerable student groups

International students may be particularly vulnerable to the impacts of COVID-19, because they are often in a more difficult financial situation than domestic students, need to comply with work visa restrictions and are often not being eligible for government subsidies (14). Due to travel restrictions, international students also suffered from being separated from their families and social networks in their home countries and often have lower social support, which makes them particularly vulnerable for social isolation and lack of support.

A cross-sectional study during the first wave of COVID-19 in spring 2020 that delivered data from students at 125 higher-education institutions in 26 countries (15) and indicated further groups of higher risk for mental health implications. The results showed that the occurrence of depressive symptoms was associated with individual factors, such as female gender, low social support, a difficult socioeconomic situation, and migration background, and COVID-19 related stressors, such as social isolation, financial problems, and academic stress (15, 16). This promotes the notion that the indirect consequences of COVID-19, e.g., due to social distancing, had more impact on students' mental health than direct effects of COVID-19, at least at the beginning of the pandemic when infection rates were still relatively low in some countries compared to subsequent waves of the pandemic.

The indirect consequences of the pandemic could also be observed in teaching. Social isolation and subjectively detrimental study conditions were found to be associated with an impaired mental health during the pandemic (8, 17, 18). Furthermore, around every second student suffered from higher workload and experienced stress due to the changes in teaching (8). These findings indicate that higher-education institutions might be able to foster their students' mental health by ensuring health-promoting study conditions.

### Implications for social networks

During the COVID-19 pandemic the typical university experience changed substantially. During university closures, students did not get to know the university setting (e.g., in their first semester), were unable to meet academic staff personally and faced technological challenges due to online teaching and remote counseling, which put them under increased pressure (5). Moreover, studying at a higher education institution is also a lifespan of establishing new relationships, and peers are of high importance for social life in early adulthood (19). During the pandemic, peers could not meet in presence, and establishing new relationships was massively hindered. This is supported by research showing that students reported decreased satisfaction with social interaction (20) and increased loneliness (17). These changes can have implications for their mental health. Loneliness, for example, is associated with diverse mental health problems such as anxiety, stress, or depressive symptoms (21). For students with mental health issues, university closures moreover meant a lack of access to the resources that they usually have at the educational setting, such as counseling (22).

### Implications for health behaviors

The phase of studying is crucial for developing health-related behaviors including risky habits (19), e.g., engaging in physical activity was affected by the pandemic, at least early in the lockdown, when sporting venues and also parks were closed (23). Interestingly, studies showed divergent responses with groups of students reporting a decrease while others reported an increase in physical activity during the first COVID-19 lockdown (23). Recent data also show changes in health risk behaviors such as substance use, but no consistent pattern was found between studies from different countries (23, 24). One explanation for changes in the engagement in health risk behaviors can be coping behavior. When students struggle with emotional problems and stress both illicit (25) and licit substance use (26) are known as unhealthy coping capacities. Such behavioral patterns were also confirmed during the COVID-19 pandemic (27). This coping behavior may be a relief in the short term, and undesirable mental health symptoms such as depressive moods, anxiety, irritability, and negative thoughts may be diminished. However, in the longer term, it aggravates them and can end in abuse or even dependence.

## Discussion and recommendations

Institutions of higher education have the potential to promote the mental health of its members and provide its services to the local community in the areas of advisory, guidance, and direction through the planning and implementation of many educational programmes. A cross-sectional survey conducted in 28 countries (28) concluded that universities can support their students during times of crises by promoting their respective coping strategies. Although the study has investigated coping with the financial crisis in 2007, this finding may serve as a good example of the impact of universities on students' coping abilities.

### Lessons learnt for teaching practice

In response to the experiences made during the online teaching phases, higher education institutions should carefully evaluate the outcomes of online or hybrid teaching. The forced experimentation with more digitized teaching and learning methods may have led to a higher variety of teaching methods on offer and better trained staff in digital teaching practices. Good practice models of digitization and online teaching could be continued and combined with on-campus teaching to allow for more flexibility in adaptation to the learners' needs. Participative development of future education could ensure that the students' voices are heard in this context.

### Re-structuring of student counseling and support services

Furthermore, high-risk groups among students and sensitive phases during university training could be identified. Universities should follow a proactive approach to contact students who might be at high risk of developing mental health issues including doubts concerning their studies (29). These could be students with migration background, with financial difficulties, or lower-educated parents, because social inequality might have increased during the COVID-19 pandemic. The pandemic could lead to even more social injustice concerning education and career opportunities in the long run and thus, to more mental health problems among young adults.

In general, psychological problems seem to occur more frequently at the beginning of university training (30) and can interact with doubts casted on the studies: students with chronic psychological conditions, above all depressive symptoms, seem to be at high risk of developing doubts and drop out of university (29, 31). Therefore, services for such vulnerable students play a crucial role. In order to improve mental health services for students, awareness raising events and programs, mental health and mindfulness training, comprehensive online portals, and peer-based support programs have been suggested (6), and such services are now even more needed to mitigate the COVID-19 impact on students' mental health.

While the current need for services may exceed the in-house capacities, linking universities to external mental health providers should also be improved and extended, and e-health services should be considered and promoted in institutional campaigns (32). E-health counseling and interventions should be made available at all institutions of higher education, because they are acceptable and effective for university student populations, and they correspond to the increasing relevance of online teaching (6). Evidence-based online health and wellbeing portals should be developed at national level for easy access within existing university digital student communication platforms.

### Importance of the health-promoting university

As Dooris and Baybutt have recently emphasized, the importance of settings is presently re-affirmed, because, to a large extent, the recovery from COVID-19 will be determined by and experienced in the settings in which we live our lives, and a settings approach is thus needed to mitigate the negative impacts of COVID-19 (33). This is especially true for universities as settings, because those institutions that have already established themselves as Health Promoting Universities managed to promote their students' and staff's resilience and mental health in the COVID-19 crisis. The higher resilience is rooted in a faster and more effective possibility to respond and adapt, because the networking and communication structures for health and social issues are already established. Most fundamental to their higher resilience, however, is that Health Promoting Universities consider their students' and staff's health as a high priority aim with relevance for the university overall functioning and performance in both teaching and research output. With putting health high on the institutional agenda, Health Promoting Universities are *per se* better equipped to respond to any health or social crisis due to the established networks within the university and with external service providers and actors. Therefore, the Health Promoting University approach should become more needed and attractive for any university that did not have this focus on health promotion yet. A whole institution approach is even more warranted since universities are rich of expertise in research, education, and student advocacy, which makes them an ideal setting for innovative approaches to university student health. However, funding must be provided both internally and externally to incentivise such development work and knowledge translation (6).

### Future research

It is important to monitor the impact of the COVID-19 pandemic on higher education students' mental health and status in order to get insights into potential long-term effects. This is relevant, because it cannot be foreseen if and how the pandemic continues and because effects of the disruption, even if transient, may have longer lasting consequences. Therefore, student surveys should include questions on health and wellbeing with special focus on mental health aspects. Moreover, data on help-seeking behaviors and experiences should be monitored from surveys, but also with data collected from counseling services in order to identify service gaps. More efforts are also needed at (inter-)national levels to coordinate such data collections and to standardize monitoring and assessment tools.

Further research should also focus on developing online platforms and tools tailored to the needs and help-seeking preferences of university students as well as on evaluating their use, student satisfaction, and effectiveness. It is critical that future researchers explore the effectiveness of health promoting strategies that are tailored toward the needs of specific student groups at high risk. At the same time, more knowledge on the multi-faceted outcomes of whole population setting-based approaches is warranted.

## Data availability statement

The original contributions presented in the study are included in the article/supplementary material, further inquiries can be directed to the corresponding authors.

## Author contributions

CS conceptualized the perspectives article and drafted the manuscript. SH and KH contributed to writing and drafting the manuscript. All authors have agreed to the final version of the manuscript.

## Conflict of interest

The authors declare that the research was conducted in the absence of any commercial or financial relationships that could be construed as a potential conflict of interest.

## Publisher's note

All claims expressed in this article are solely those of the authors and do not necessarily represent those of their affiliated organizations, or those of the publisher, the editors and the reviewers. Any product that may be evaluated in this article, or claim that may be made by its manufacturer, is not guaranteed or endorsed by the publisher.
